# Supplementary material for: Electrical synapses convey orientation selectivity in the mouse retina
Source: Nat Commun. 2017 Dec 11;8:2025. doi: 10.1038/s41467-017-01980-9 (PMC5725423; doi:10.1038/s41467-017-01980-9)
Supplement: Supplementary file 1 — Supplementary Information [file 41467_2017_1980_MOESM1_ESM.pdf]

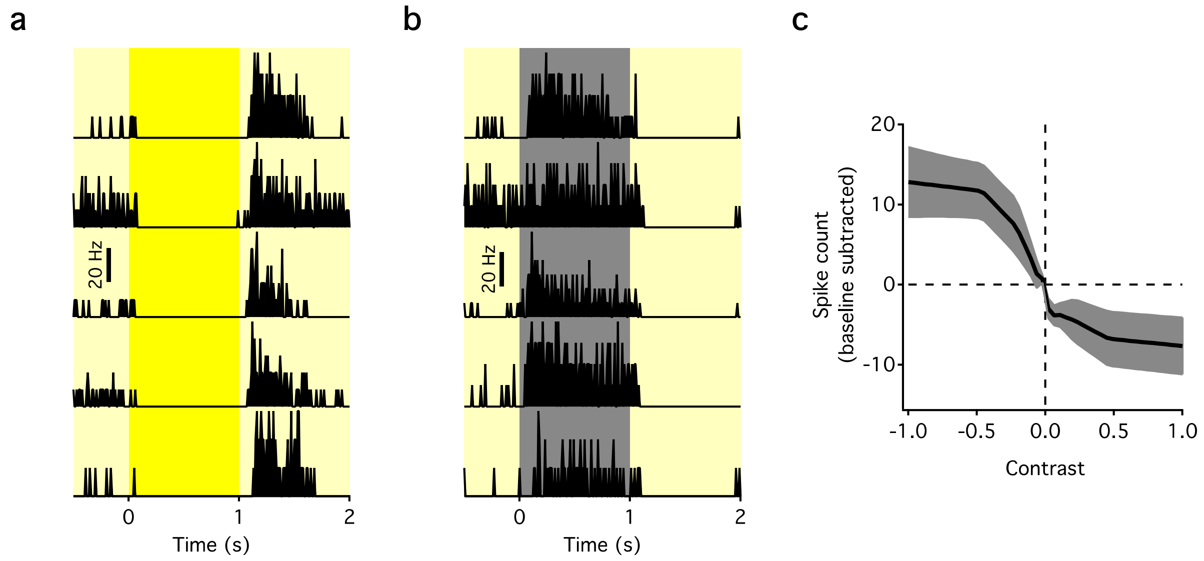

Supplementary Figure 1 Contrast response function of OFF OS RGCs. (**a**) Peristimulus time histograms (PSTHs) of 100% positive Weber contrast step responses of 5 representative OFF OS RGCs. PSTHs are calculated across 5 trials for each cell. A background mean illumination of 500 R*/rod/s is used. (**b**) Same as in (**a**) but 100% negative Weber contrast steps are used. (**c**) Contrast response function of OFF OS RGCs. Shaded region indicates SEM across n = 5 cells.

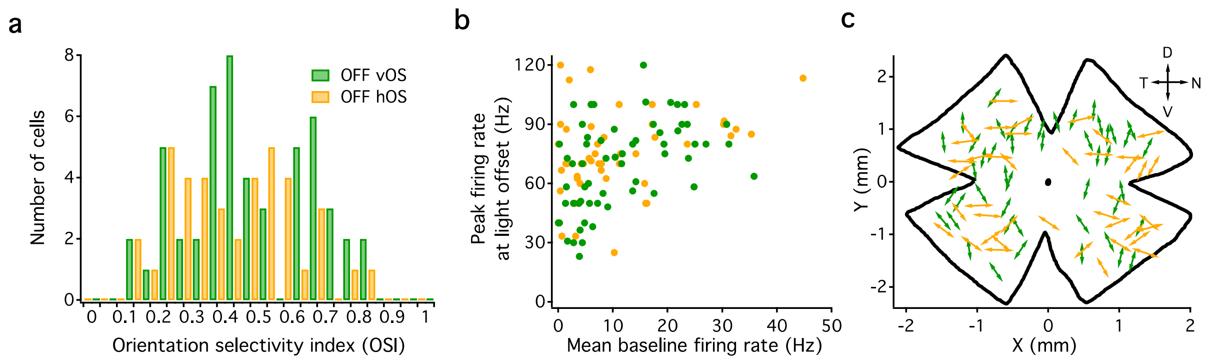

Supplementary Figure 2 OFF vOS and OFF hOS RGCs have similar light response profiles. (**a**) Peak firing rate at light offset plotted against mean baseline firing rate for OFF vOS (n = 52) and OFF hOS (n = 40) RGCs. (**b**) Histogram of OSI of OFF vOS (n = 52) and OFF hOS RGCs (n = 40). (**c**) Positional map of the OFF OS RGCs (n = 92) in the whole retina.

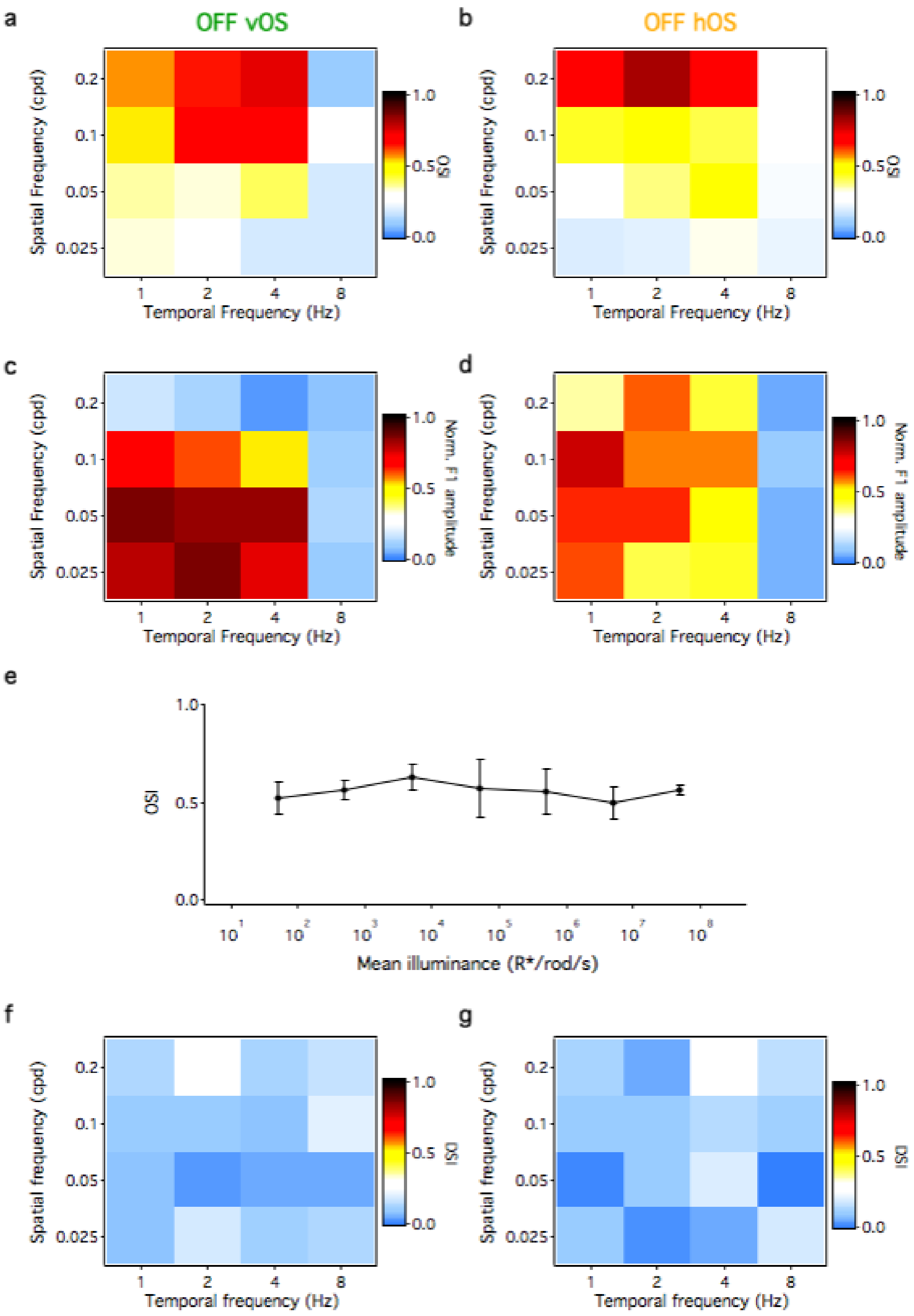

Supplementary Figure 3 Spatiotemporal tuning of OFF OS RGCs to drifting gratings. (**a**, **b**) Heat maps of OSI of OFF vOS RGCs (n=3) (**a**) and OFF hOS RGCs (n=3) (**b**). (**c**, **d**) Heat maps of normalized F1 response amplitudes of OFF vOS RGCs (n=3) (**c**) and OFF hOS RGCs (n=3) (**d**). (**e**) OSIs of drifting grating responses of OFF OS RGCs across 7 different mean background illumination levels. Error bars indicate SEM across n=8 cells. (**f**, **g**) Heat maps of DSI of same OFF vOS RGCs (n=3) (**f**) and OFF hOS RGCs (n=3) (**g**) as in (**c**) and (**d**).

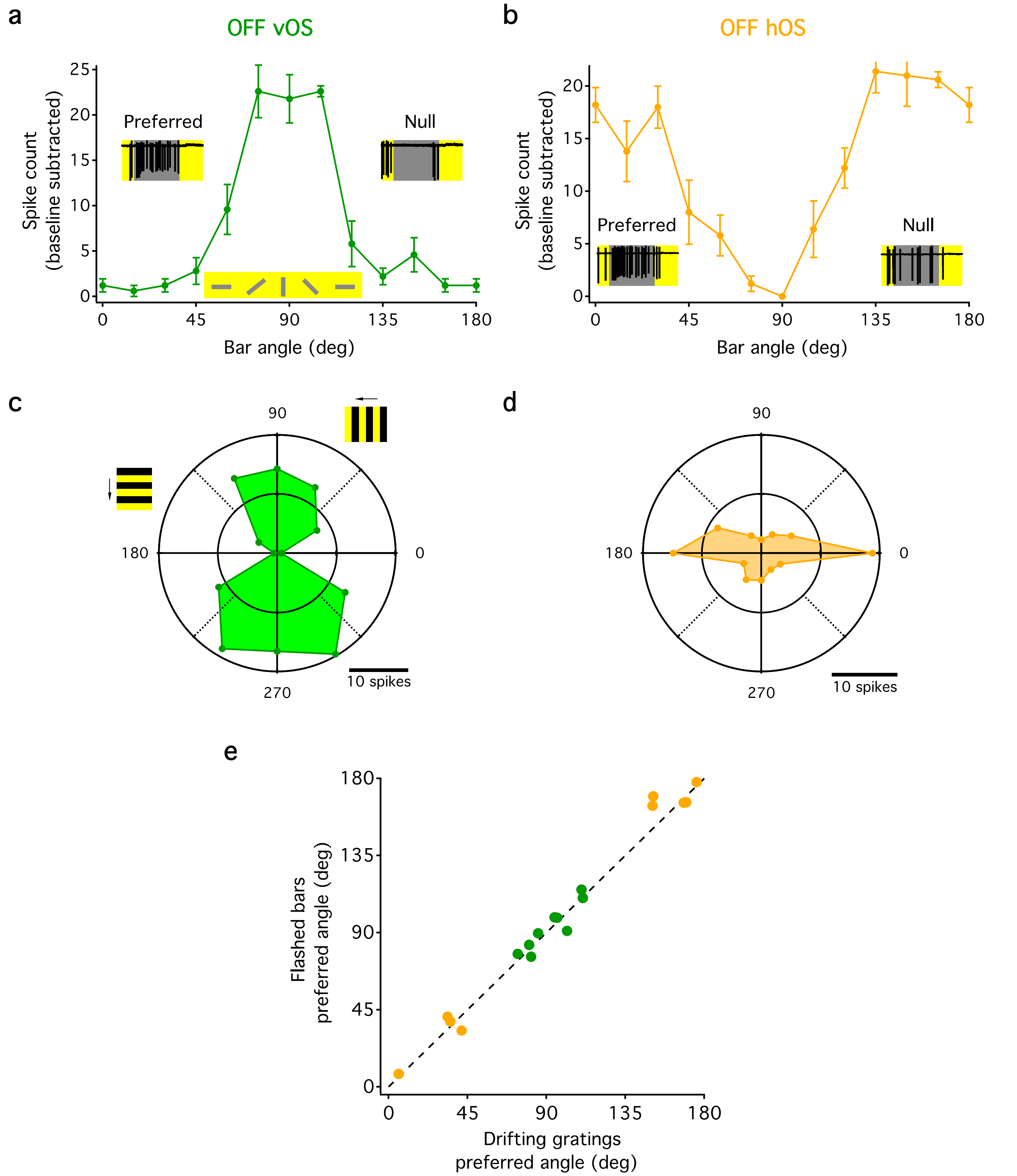

Supplementary Figure 4 Responses of OFF OS RGCs to flashed bars. (**a**, **b**) Spike responses of an (**a**) OFF vOS RGC and an (**b**) OFF hOS RGC to oriented dark bar stimuli (800 μm x 50 μm, 12 angles) from a mean illuminance. Preferred and null orientation responses are shown in insets. Error bars indicate SEM across five trials along each orientation. (**c**, **d**) Responses of the same RGCs in (**a**) and (**b**) to drifting gratings. (**e**) Scatter plot of recorded OS angles in response to flashed bars versus OS angles recorded in responses to drifting gratings.

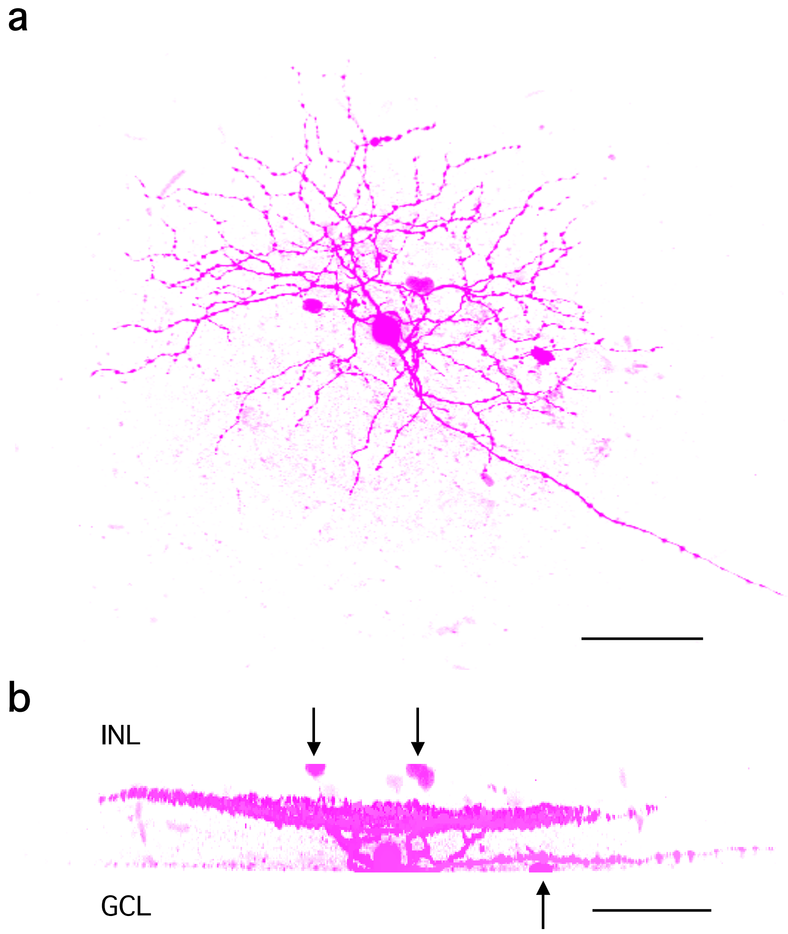

Supplementary Figure 5 Morphological coupling of OFF hOS RGCs. (**a**) Neurobiotin tracer filled image of an OFF hOS RGC. (**b**) Side view of the cell in (**a**) showing weak coupling to cells in GCL and INL. Scale bars = 50 μm.

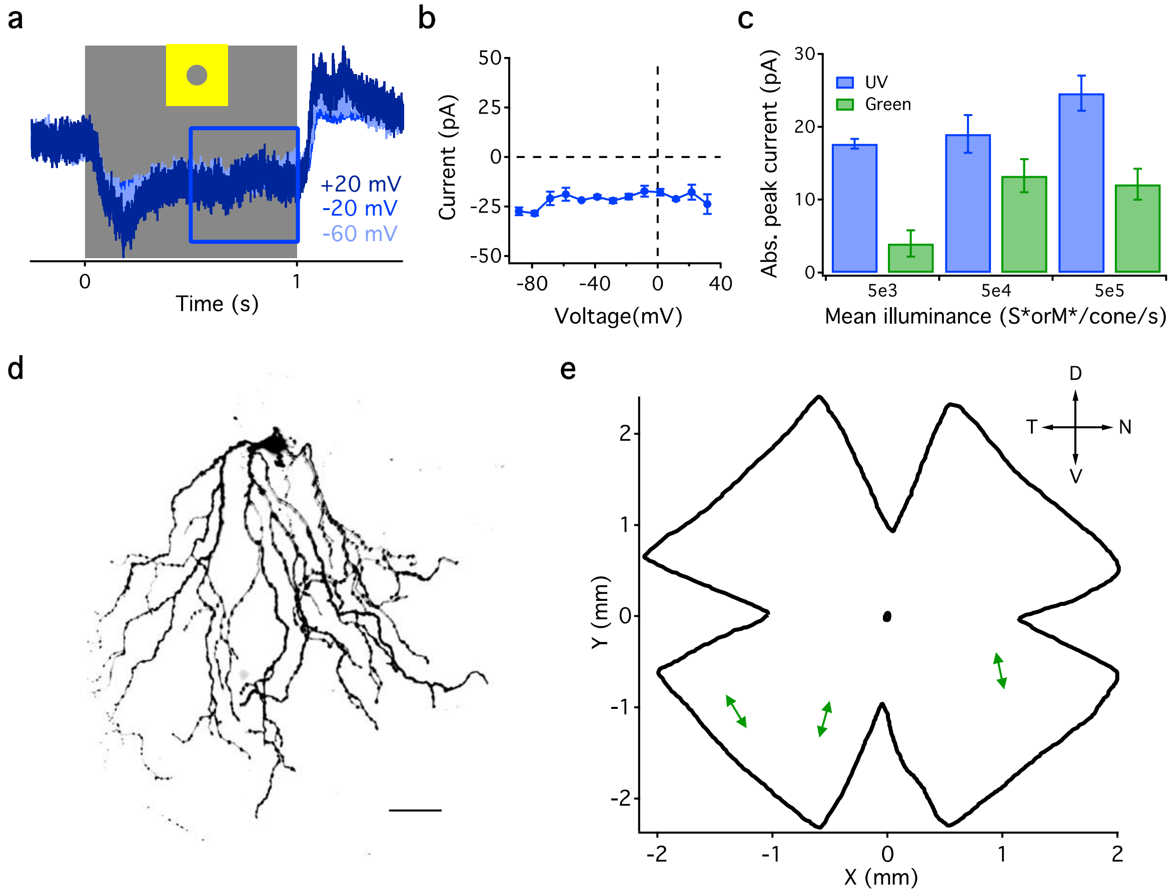

Supplementary Figure 6 Gap junction currents dominate the responses of OFF OS RGCs to negative contrast steps across luminance and color conditions. (**a**) Currents evoked in an OFF OS RGC by a 90% negative contrast UV light step (200 μm diameter) from 500,000S* mean illuminance measured across a range of holding voltages. Gray rectangle indicates stimulus. (**b**) IV relationship for the stimulus in (**a**). Error bars indicate SEM across 4 trials. Peak currents were measured in 500–1000ms temporal window as indicated by blue rectangle in (**a**). (**c**) Absolute peak currents measured for 90% negative contrast UV and green light steps at 3 different mean illumination levels. Error bars indicate SEM across n = 3 cells. (**d**) Dendritic morphology of a recorded OFF vOS cell. Scale bar = 50 μm. (**e**) Position map of recorded OFF vOS RGCs.

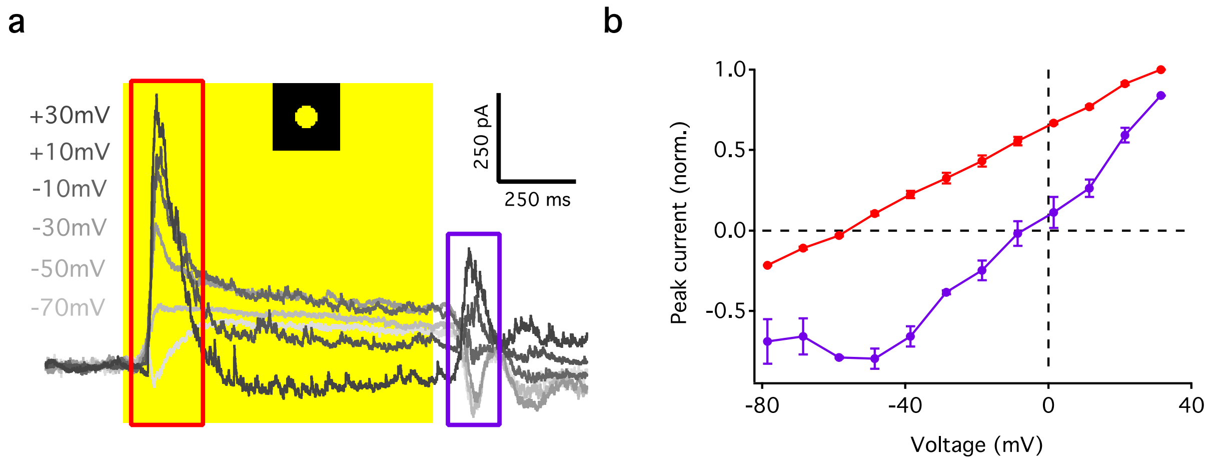

Supplementary Figure 7 Removal of presynaptic inhibition reveals excitatory synaptic inputs to OFF OS RGCs. (**a**) Current evoked in an OFF OS RGC in presence of bath applied gabazine by a light step (200 μm diameter) from darkness measured across a range of holding voltages. Yellow rectangle indicates light stimulus. (**b**) IV relationship for the stimulus in (**a**). Error bars indicate SEM across n = 4 cells. Peak currents were measured in the temporal windows (0-200ms, red rectangle and 1000-1200ms, purple rectangle) indicated in (**a**).

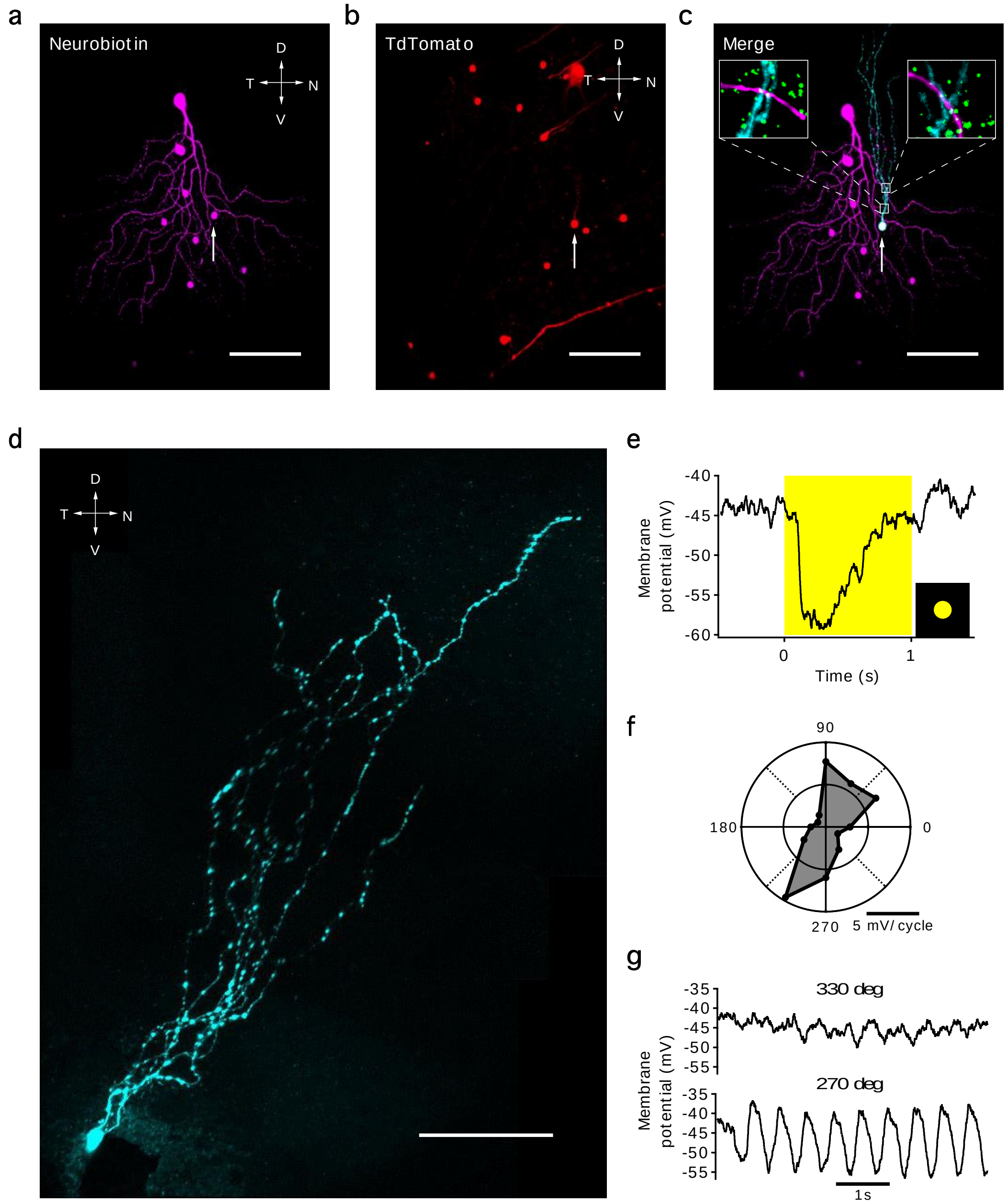

Supplementary Figure 8 ACs coupled to OFF OS RGCs are OS. (**a**) Morphology of an OFF vOS RGC filled with neurobiotin in Etv1 transgenic retina. Coupled amacrine cells are also neurobiotin positive. (**b**) TdTomato labeled cell somas in Etv1 transgenic retina. (**c**) TdTomato fluorescence is present in the soma of one of the ACs coupled to OFF vOS RGC. The AC is traced and pseudocolored cyan. Insets show existence of Cx36 puncta (green) at crossings between ganglion cell (magenta) and amacrine cell (cyan) dendrites. Scale bars = 50 μm. (**d**) Morphology of a traced Etv1 OFF OS AC filled with Alexa 488 dye. Scale bar = 100 μm. (**e**) Response of Etv1 OFF OS AC to a 1s flash of 200 μm diameter circular light spot from darkness. Yellow rectangle indicates light stimulus. (**f**) Polar plot of drifting grating responses of Etv1 OFF OS AC. (**g**) Raw traces of recorded membrane potentials of the same cell in (**f**) along preferred and null orientations.

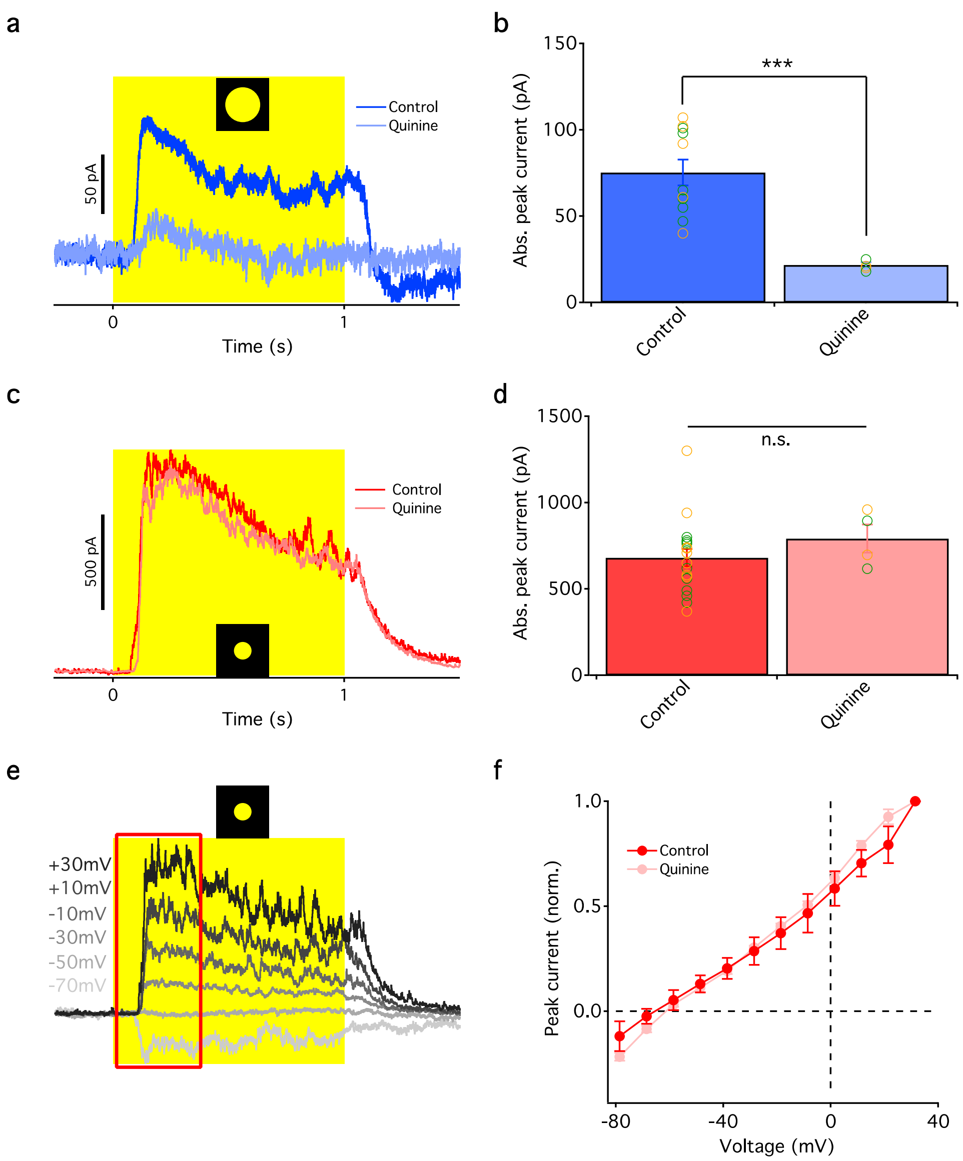

Supplementary Figure 9 Pharmacological block of Cx36 mediated gap junction currents by quinine. (**a**) Effect of quinine on light step responses (1200 µm diameter) of an example OFF OS RGC from darkness. Cell is voltage clamped at E_{Cl}. Yellow rectangle indicates light stimulus. (**b**) Absolute peak currents evoked by a 1200 µm diameter spot of light in OFF OS RGCs in control situation and intracellular application of quinine. *** $p < 0.001$. (**c**) Effect of quinine on light step responses (200 µm diameter) of an example OFF OS RGC from darkness. Cell is voltage clamped at E_{Cat}. (**d**) Absolute peak currents evoked by a 200 µm diameter spot of light in OFF OS RGCs in control situation and intracellular application of quinine. (**e**) Current evoked in an OFF OS RGC by a light step (200 µm diameter) from darkness measured across a range of holding voltages in the presence of quinine. (**f**) IV relationship for the stimulus in (**e**) indicated by light red trace. Error bars indicate SEM across n = 4 cells. Dark red trace is same as in Fig. 4b. Peak currents were measured in a 0-200ms temporal window after light onset indicated by red rectangle.
